# Reactive oxygen species modulator-1 (Romo1) predicts unfavorable prognosis in colorectal cancer patients

**DOI:** 10.1371/journal.pone.0176834

**Published:** 2017-05-04

**Authors:** Hong Jun Kim, Min Jee Jo, Bo Ram Kim, Jung Lim Kim, Yoon A. Jeong, Yoo Jin Na, Seong Hye Park, Suk-young Lee, Dae-Hee Lee, Hye Seung Lee, Baek-hui Kim, Sun Il Lee, Byung Wook Min, Young Do Yoo, Sang Cheul Oh

**Affiliations:** 1 Division of Oncology/Hematology, Department of Internal Medicine, College of Medicine, Korea University, Seoul, Republic of Korea; 2 Graduate School of Medicine, Korea University College of Medicine, Korea University, Seoul, Republic of Korea; 3 Department of Pathology, Korea University Guro Hospital, Korea University College of Medicine, Seoul, Republic of Korea; 4 Department of Surgery, Korea University Guro Hospital, Korea University College of Medicine, Seoul, Republic of Korea; 5 Laboratory of Molecular Cell Biology, Graduate School of Medicine, Korea University, Seoul, Republic of Korea; Sapporo Ika Daigaku, JAPAN

## Abstract

**Background:**

Reactive oxygen species modulator-1 (Romo1) is a novel protein that has been reported to be crucial for cancer cell proliferation and invasion. However, its clinical implications in colorectal cancer patients are not well-known. For the first time, we investigated the association between Romo1 and the clinical outcomes of colorectal cancer patients.

**Study:**

We examined Romo1 expression in resected tumor tissues immunohistochemically and assessed it with histological scores. We conducted survival analyses for patients who had curative resection (n = 190) in accordance with clinical parameters including level of Romo1 expression, and we examined the association between Romo1 expression and cell invasion using Matrigel invasion assay in colorectal cancer cells.

**Results:**

We observed significantly longer mean disease-free survival in the low Romo1 group compared with the high Romo1 group (161 vs 127.6 months, p = 0.035), and the median overall survival of the low Romo1 group was significantly longer than that of the high Romo1 group (196.9 vs 171.3 months, p = 0.036). Cell invasiveness decreased in the Romo1 knockdown colorectal cancer cells in contrast to the controlled cells. Romo1 overexpression in tumor tissue was associated with a high lymph node ratio between the metastatic and examined lymph nodes (p = 0.025).

**Conclusions:**

Romo1 overexpression in tumor tissue was significantly associated with survival in curatively resected colorectal cancer patients, suggesting Romo1 expression as a potential adverse prognostic marker. Increased Romo1 expression was found to be associated with high lymph node ratio. Cancer invasiveness appeared to be a key reason for the poor survival related to highly expressed Romo1.

## 1. Introduction

Colorectal cancer (CRC) accounts for approximately 10% of all incident cancers, and there are approximately 600,000 CRC deaths annually worldwide[1]. In the United States, CRC is one of the leading causes of cancer mortality, accounting for nearly 10% of deaths[2]. In south Korea, CRC accounts for about 14% of all prevalent cancers, and about 8000 CRC deaths occur annually (nearly 11% of all cancer related deaths)[3]. Surgical resection is the primary treatment for CRC, and in the early stage, satisfactory survival rates can only be expected after curative resection. In cases of recurrent, metastatic, or locally advanced CRC, patients are treated with non-selective cytotoxic chemo-agents. Recently, targeted agents including epidermal growth factor receptor inhibitor (cetuximab), vascular endothelial growth factor (VEGF) A inhibitor (bevacizumab), and multi-kinase inhibitor (regorafenib) have been introduced for treating recurrent or metastatic CRC and have shown preferable results[4].

Compared with targeted agents, preexisting cytotoxic agents deliver toxic effects to not only cancer but also normal cells, such that these agents bring severe systemic adverse effects and have limited treatment effects. However, newly developed targeted agents could minimize side effects and maximize treatment effects by targeting not normal cells but cancer cells only[5, 6]. A better comprehension of the mechanisms involved in cancer development and metastasis could improve the management of CRC and be followed by the development of new agents. Not only that, these studies also could also bring new discovery and clinical application of biomarkers for detecting patients who are expected to have the best responses to specific treatments.

Reactive oxygen species modulator-1 (Romo1) is a novel mitochondrial protein that was first cloned in 2006 as a newly emerged gene in cancer tissues that had become resistant during chemotherapy[7]. Upregulation of Romo1, which is the key regulator of mitochondrial reactive oxygen species (ROS) release[8], was observed in various cancer cells and reported to be crucial for cancer cell proliferation and invasion[9, 10]. Romo1-induced ROS is reported to contribute cancer cell proliferation through extracellular signal-regulated kinases (ERK) activation and constitutive activation of nuclear factor kappa B (NF-kB)[9, 11]. Furthermore, the mechanism by which Romo1 affects cancer invasiveness is reported to be associated with epithelial-mesenchymal transition (EMT) markers and the NF-kB signaling pathway[12, 13]. In addition to *in vitro* studies, a small number of studies have demonstrated clinical applications of Romo1. A previous study suggested that Romo1 overexpression induces tumor invasion and contributes to poor prognosis in patients with hepatocellular carcinoma (HCC)[14]. Other studies reported that Romo1 as a potential prognostic or diagnostic biomarker in patients with non-small cell lung cancer (NSCLC)[15–17]. However, there has been no study concerning the clinical implications of Romo1 in patients with CRC.

In the present study, we investigated the association of Romo1 expression with survival rates and clinical parameters in CRC patients.

## 2. Materials and methods

### 2.1 Study patients and specimens

We collected formalin-fixed, paraffin-embedded tumor samples from CRC patients who underwent surgical resection from 1998 to 2000 at Korea University’s Guro Hospital. We excluded patients who had died within one month of surgery or who had received neoadjuvant chemotherapy or radiotherapy to reduce bias in the survival analysis, and we excluded five patients whose specimens were not suitable for IHC (immunohistochemical) staining, for a total of 208 patients enrolled. One hundred ninety patients had no distant metastatic lesions and underwent curative surgical resection; the other 18 had distant metastatic lesions but underwent surgical resection because of signs of obstruction. We collected the specimens after surgical resection and confirmed pathologic staging according to the AJCC (American Joint Committee on Cancer) 6^th^ edition. We obtained the clinical data by retrospectively reviewing medical records. We performed this study with the permission of the Guro Hospital Institutional Review Board (KUGH15356-001) but no informed consent was obtained because the data were analyzed anonymously.

### 2.2 Romo1 scoring with IHC staining

We used the collected paraffin blocks as donor blocks to make tissue microarray (TMA) recipient blocks. In each donor block, we chose and marked morphologically representative areas on their respective H&E slides. We took a tissue core of 0.6mm in diameter from each donor block using a cylindrical tissue puncher (Micro Digital Co., Seoul, Korea) and transferred each core into the hole on the recipient paraffin block.

We used IHC staining to demonstrate the Romo1 expression. After deparaffinization with xylene, we incubated the TMA slides with peroxidase blocking reagent (3% H_2_O_2_ diluted with dextrose water) for 15min to erase endogenous peroxidase activity. Then, we retrieved the antigens by heating samples at 95°C for 20min and cooling them for 10min. After universal block with blocking buffer, we incubated the TMA slides with Romo1 antibody (OriGene Technologies, Rockville, USA) at a 1:200 dilution. We then developed samples with 3,3’-diaminobenzidine chromogen solution for 7min, followed by counterstaining with hematoxylin and dehydrating. Negative control slides without primary antibody were included.

Two investigators (Baek-hui Kim^3^and Hong Jun Kim) who were unaware of the clinical information independently evaluated Romo1 staining under a light microscope at 200×; when they observed cytoplasmic staining, they recorded the stained cells as positive. The staining intensity was divided into 4 grades (intensity score 0~3), and the staining percentage of positive cells was divided into 10 grades (percentage score 1~10). The readings between the two investigators were about 80% identical. In case the results were not equal between the two readers, we asked a third party to conduct an inspection. Romo1 expression was calculated by multiplying the percentage score by the intensity score (possible range, 0–30).

### 2.3 Reagents, antibodies, small interfering RNA (siRNA), short hairpin RNA (shRNA), and plasmid DNA

We obtained RPMI 1640 medium, fetal bovine serum (FBS), and antibiotics from Gibco (Carlsbad, CA, USA), and obtained antibodies including Romo1 (OriGene Technologies, Rockville, USA) and β-actin (Sigma, St. Louis, MO, USA) as well. We obtained Romo1 siRNA, Romo1 shRNA, control siRNA, control shRNA, and plasmid DNA (pFlag-c1 (control) and pFlag-c1 Romo1) from Santa Cruz Biotechnology (Santa Cruz, CA, USA).

### 2.4 Cell culture and transfection

We purchased the human CRC cell lines (HCT116 and DLD-1) from the American Type Culture Collection (ATCC, Manassas, VA, USA) and maintained them according to the ATCC’s instructions. We grew all cell lines in RPMI 1640 supplemented with 10% FBS and 1-glutamine and grown in a 37°C humidified chamber at 5% CO_2_. We transfected the Romo1 siRNA, Romo1 shRNA, control siRNA, and control shRNA using Lipofectamine RNAiMAX transfection reagent (Invitrogen). We transfected plasmid DNA into CRC cells using Lipofectamine 2000 transfection reagent (Invitrogen).

### 2.5 Western blotting

We lysed the cells in RIPA buffer (50mM Tris, 150 mM NaCl, 1% Triton X-100, 0.1% SDS and 1% Na-deoxycholate [pH 7.4]) with phosphatase inhibitor and proteases cocktails and subjected them to SDS-PAGE. Then, we transferred the cells onto nitrocellulose membranes (GE Healthcare Life Sciences, Logan, UT USA) and blocked them with TBS that contained 0.2% Tween-20 and 5% skim milk; we incubated them with the primary antibody and then with the horseradish peroxidase-labeled secondary antibody, and detected the signals by X-ray film.

### 2.6 Cell viability assay, wound healing assay, and Matrigel invasion assay

We grew the CRC cells in a 96-well plate at 1x10^4^ cells/well. We transfected the cell lines (HCT116 and DLD-1) with Romo1 shRNA or control shRNA, with Romo1 siRNA or control siRNA, and with pFlag-c1 (con) or pFlag-c1 Romo1. At 24h after transfection, we added 25 μl of 3-(4,5-dimethylthiazol-2-yl)-2,5-diphenyltetrazolium bromide (MTT) (5 mg/ml) (Sigma-Aldrich Co.LLC, St. Louis, MO, USA) to each well and incubated the plates for 4h at 37°C. We then aspirated the MTT solution in the medium and added 150 μl of dimethyl sulfoxide before we measured the absorbance at 550nm.

For the wound healing assay, we seeded cells at 5x10^5^ cells/well in 12-well plates. At 100% confluence, we made two parallel wounds using a plastic pipette tip and then grew the cells in culture medium with 5% FBS. We collected images of the wound at 0h, 24h, and 48h using a microscope and quantified the migration rate by measuring the length between the wound edges. We repeated this assay two times independently.

For the Matrigel invasion assay, we seeded 3x10^5^ cells/well in the upper chamber, which was coated with Matrigel (BD biosciences). After 48 h at 37°C in 5% CO_2_, we stained the cells that were present on the lower surface of the insert with Diff-Quik Stain kit (Biochemical Sciences, Inc., Swedesboro, NJ, USA). We counted the cells invaded through the Matrigel-coated membrane by microscopy.

### 2.7 Statistical analyses

The clinical outcomes we assessed were disease-free survival (DFS) and overall survival (OS); we defined DFS as the period after surgical resection during which there were no signs of the disease or death from any cause and OS as the period from surgical resection to death from any cause; we censored data for cases without cancer recurrence or death at the point of the last follow-up. We assessed the associations between survival and the clinical parameters by univariate analysis with the log rank test and by multivariate Cox’s proportional hazard regression. For the multivariate analysis, we incorporated the clinical variables into the model in a stepwise manner. We determined the cut-off level for low versus high Romo1 expression after we analyzed the p values on the log rank test between the two groups from all possibilities. As a result, we identified the optimal cut-off level (the one that showed the lowest p value on the log rank test) as 19. We made survival curves using the Kaplan-Meier method and assessed the relationship between the clinicopathological CRC parameters and the Romo1 expression levels using the Chi-square test. Using the Mann-Whitney U test, we measured the associations between the Romo1 expression levels in both patients groups by clinical parameters, considering p values less than 0.05 to be significant. We conducted all statistical analyses using SPSS version 20.0 for Windows (SPSS, Chicago, IL, USA).

## 3. Results

### 3.1 Patient characteristics

The characteristics of the patients are shown in Table 1. The entire study population was Korean, and the median age was 58 years (range, 26 to 84 years). One hundred and fifteen patients (55%) were male, and 171 patients were under 70 years of age. Eighty-six patients (41%) were stage II, 76 patients (37%) were stage III, and 18 patients (9%) were stage IV. Among patients who underwent curative surgical resection (n = 190), 135 patients had adjuvant chemotherapy, and we used the same 5-fluorouracil/leucovorin (5FU/LV) regimen for all patients (n = 135) receiving adjuvant chemotherapy. Among patients with stage IV CRC (n = 18), 10 had palliative systemic chemotherapy; the chemotherapy regimens used for first-line treatment were oxaliplatin/5FU (n = 6) and 5FU (n = 4). Eighty-five patients (41%) had well-differentiated cancer and 123 (59%) had moderately to poorly differentiated cancer. Given that well-differentiated cancers account for approximately 5–6% of the global CRC population[18], the proportion of well-differentiated cancer in our cohort (41%) is much higher than expected. However, data from 2230 CRC patients in Korea[19] show that well-differentiated cancers constitute about 41%, which is consistent with our data. Ninety patients (43%) had cancer with lymph node metastasis, and 57 (27%) had lymph node ratios (LNR) over 0.1 between the metastatic and examined lymph nodes.

**Table 1:** Association between Romo1 and clinicopathologic parameters.

	No. of patients (%)	Average score of Romo1	P value
All	208(100)	13.6	
Gender			0.581
Female	93(45)	13.9	
Male	115(55)	13.4	
Age			0.845
<70	171(82)	13.6	
≥70	37(18)	13.9	
Stage			0.869
I	28(13)	13.7	
II	86(41)	13.2	
III	76(37)	13.8	
IV	18(9)	14.9	
N stage			0.547
N0	118(57)	13.4	
≥N1	90(43)	14.0	
LNR			0.025
<0.1	151(73)	12.9	
≥0.1	57(27)	15.7	
T stage			0.644
T1,T2	35(17)	14.5	
T3,T4	173(83)	13.5	
Lymphatic invasion			0.043
Negative	87(42)	12.2	
Positive	121(58)	14.7	
Vascular invasion			0.979
Negative	132(63)	13.4	
Positive	76(37)	13.9	
Tumor differentiation			0.836
Well	85(41)	13.4	
Mod-poor	123(59)	13.8	

N, lymph node; LNR, lymph node ratio; T, tumor

### 3.2 Romo1 protein expression in CRC tissues

**Fig 1:**
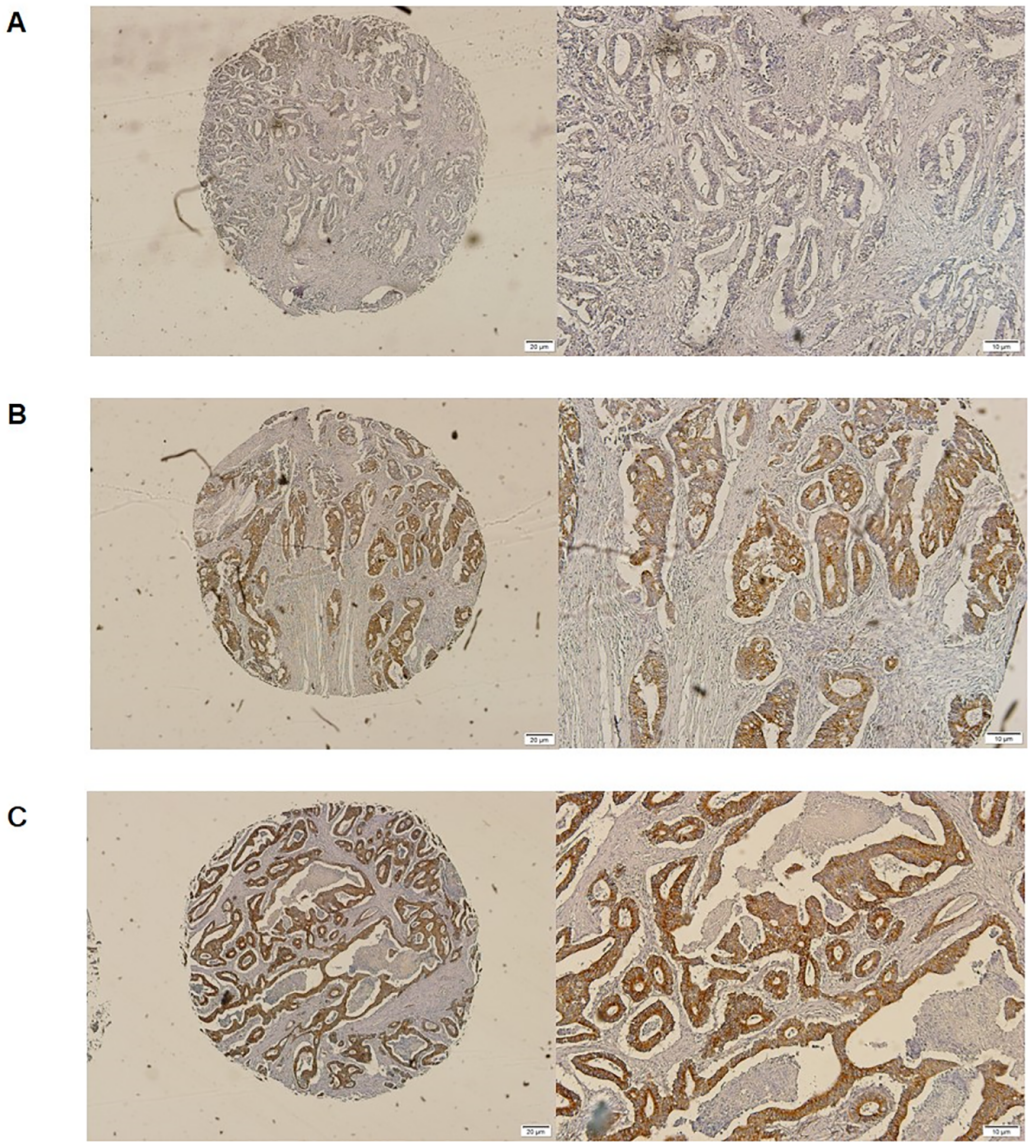
Representative tissue samples showing different levels of Romo1 expression. Romo1 was detected in the cytoplasm of cancer cells. (A) Romo1 expression level of 0. Magnification, x40 and x100; (B) Romo1 expression level of 12. Magnification, x40 and x100; (C) Romo1 expression level of 30. Magnification, x40 and x100.

Representative tissue samples showing different levels of Romo1 expression are shown in Fig 1. Romo1 protein is essentially localized in the cytoplasm of cancer cells, and the Romo1 levels were not normally distributed (average: 13.6, range, 0 to 30).

### 3.3 Romo1 expression and clinicopathologic parameters

To identify the clinical factors that are related to Romo1, we compared the Romo1 levels between the groups by individual clinical parameters. Romo1 expression was related to LNR and lymphatic invasion of primary tumors (p values were 0.025 and 0.043 respectively), but not other parameters including age, gender, N stage, tumor size, and tumor differentiation (Table 1).

Using our cut-off Romo1 level of 19, we classified 156 patients in the low Romo1 group and 52 in the high Romo1 group. When we analyzed the values for the clinical parameters in these two patient groups, only LNR and lymphatic invasion were associated with Romo1 level significantly (p values were 0.015 and 0.020 respectively) (Table 2).

**Table 2:** The proportions of patients with low and high ROMO1 expression according to clinicopathologic parameters.

	No. of patients (%)	ROMO1 expression		P value
		Low (<19)	High (≥19)	
All	208(100)	156(75)	52(25)	
Gender				0.573
Female	93(45)	68(73)	25(27)	
Male	115(55)	88(77)	27(23)	
Age				0.346
<70	171(82)	126(74)	45(26)	
≥70	37(18)	30(81)	7(19)	
Stage				0.334
I	28(13)	18(64)	10(36)	
II	86(41)	72(84)	14(16)	
III	76(37)	56(74)	20(26)	
IV	18(9)	10(56)	8(44)	
N stage				0.258
N0	118(57)	92(78)	26(22)	
≥N1	90(43)	64(71)	26(29)	
LNR				0.015
<0.1	151(73)	120(79)	31(21)	
≥0.1	57(27)	36(63)	21(37)	
T stage				0.069
T1,T2	35(17)	22(63)	13(37)	
T3,T4	173(83)	134(77)	39(23)	
Lymphatic invasion				0.020
Negative	87(42)	81(94)	6(6)	
Positive	121(58)	75(62)	46(38)	
Vascular invasion				0.657
Negative	132(63)	100(75)	32(25)	
Positive	76(37)	56(74)	20(26)	
Tumor differentiation				0.807
Well	85(41)	63(74)	22(26)	
Mod-poor	123(59)	93(76)	30(24)	

N, lymph node; LNR, lymph node ratio; T, tumor

### 3.4 Survival and prognostic factors in patients who had curative resection (n = 190)

Recent studies have revealed that LNR is superior to the previous N staging system in predicting the prognosis of a number of cancers including CRC[20, 21]. In our cohort, overexpressed Romo1 was related to high LNR and lymphatic invasion, suggesting that upregulating Romo1 might have influence on the clinical outcomes in CRC patients after surgery. To determine this possibility, we evaluated the prognostic value of Romo1 in our cohort. The overall median follow-up time from surgery was 61 months (range, 2–216 months), and the median time among surviving patients was 63.5 months (range, 2–216 months). At the time of analysis, 18 deaths and 43 recurrences had occurred, and 50 patients had had recurrences or died. The median follow-up time from recurrence was 11 months (range, 0–208 months). Nine of the patients with recurrence showed local recurrence, 38 had distant metastasis, and 4 had both. Eleven patients received surgical treatment after recurrence, 30 had chemotherapy, and 7 received both; the chemotherapy regimens were oxaliplatin/5FU (n = 19) and 5FU (n = 11). Four of the recurred patients received chemotherapy after surgical treatment and showed no evidence of disease. One hundred and forty patients had no recurrence and were still alive.

Survival data with reference to clinical parameters are summarized in Table 3. The mean DFS was 153.8 months (95% CI: 139.5–168.1 months). By univariate analysis with the log rank test, patients who had no lymph node metastasis (p<0.001) and lower LNRs (p<0.001) had better DFS. In addition, patients in the low Romo1 group had significantly better DFS (161.0 vs 127.6 months, p = 0.035). By multivariate analysis with Cox’s proportional hazard regression model, lymph node metastasis (HR: 2.731, 95% CI: 1.525–4.888), higher LNR (HR: 3.582, 95% CI: 2.048–6.265), and high Romo1 expression (HR: 2.133, 95% CI: 1.167–3.896) were significantly related to poorer DFS.

**Table 3:** Survival analyses for patients who underwent curative resection.

	No of patients (%)	Disease-free survival (DFS)			Overall survival (OS)		
		Mean DFS (months)	Univariate analysis p	Multivariate analysis adjusted HR(95% CI)	Mean OS (months)	Univariate analysis p	Multivariate analysis adjusted HR(95% CI)
All	190(100)	153.8			191.6		
Gender			0.226			0.169	
Female	85(45)	164.8			201.4		
Male	105(55)	138.2			183.6		
Age			0.773			0.544	
<70	154(81)	152			192.9		
≥70	36(19)	141			156.1		
N stage			<0.001	2.731(1.525~4.888)		0.001	4.671(1.655~13.185)
N0	114(60)	174.8			204.0		
≥N1	76(40)	121.3			171.9		
LNR			<0.001	3.582(2.048~6.265)		0.002	4.009(1.585~10.141)
<0.1	143(75)	170.4			199.5		
≥0.1	47(25)	99.9			165.1		
T stage			0.053			0.455	
T1,T2	35(18)	177.7			195.9		
T3,T4	155(82)	146.6			190.2		
Tumor differentiation			0.087			0.062	
Well	79(42)	168.7			203.3		
Mod-poor	111(58)	142.1			182.5		
Romo1			0.035	2.133(1.167~3.896)		0.036	2.735(1.029~7.271)
Low	146(77)	161			196.9		
High	44(23)	127.6			171.3		

N, lymph node; LNR, lymph node ratio; T, tumor

**Fig 2:**
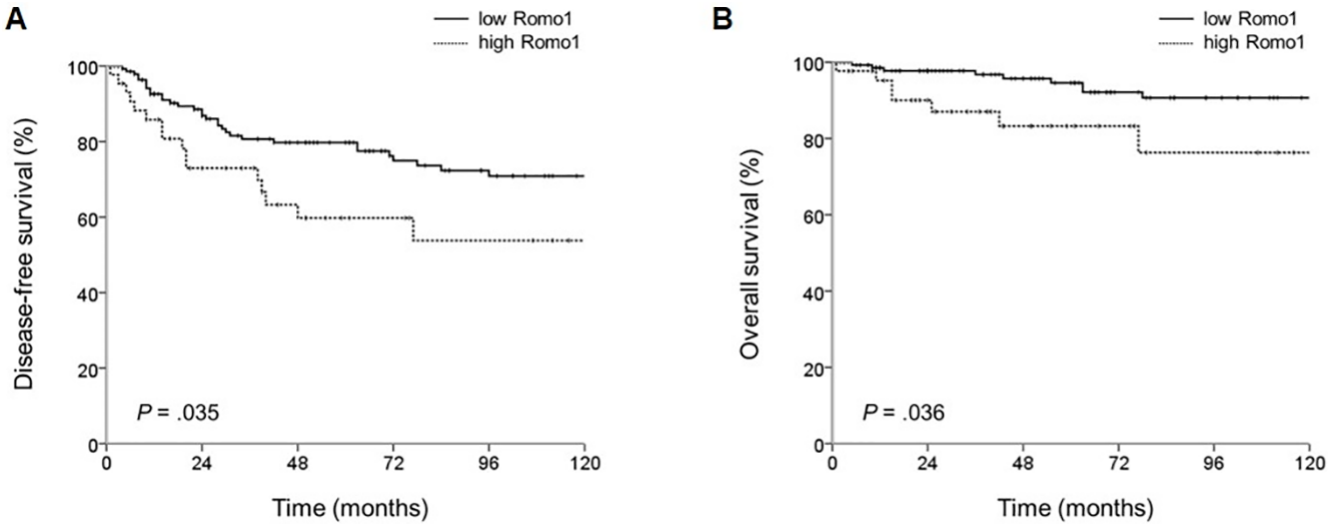
**The cumulative disease-free (A) and overall (B) survival rates among patients who had curative resection by Romo1 expression levels.** P values were defined by the log rank test.

The mean OS of this cohort was 191.6 months (95% CI: 180.9–202.2 months). Univariate analysis with the log rank test showed that patients who had no lymph node metastasis (p = 0.001) and lower LNR (p = 0.002) had better OS. Patients in the low Romo1 group had significantly better OS (196.9 vs 171.3 months, p = 0.036). By multivariate analysis with Cox’s proportional hazard regression model, lymph node metastasis (HR: 4.671, 95% CI: 1.655–13.185), higher LNR(HR: 4.009, 95% CI: 1.585–10.141), and high Romo1 expression (HR: 2.735. 95% CI: 1.029–7.271) were significantly associated with poorer OS. In Kaplan-Meier survival curves, patients who had high Romo1 expression had poorer DFS and OS (Fig 2).

### 3.5 Survival and prognostic factors in the whole cohort including stage IV CRC patients (n = 208)

We had 18 patients with stage IV CRC who had undergone surgical resection because of obstruction, and we evaluated the prognostic value of Romo1 in the whole cohort including this group. Because other cancer lesions remained after surgery, we could not define DFS in this group. The median follow-up time from surgery was 52 months (range, 2–216 months), and at the time of the last follow-up, 23 deaths had occurred.

**Fig 3:**
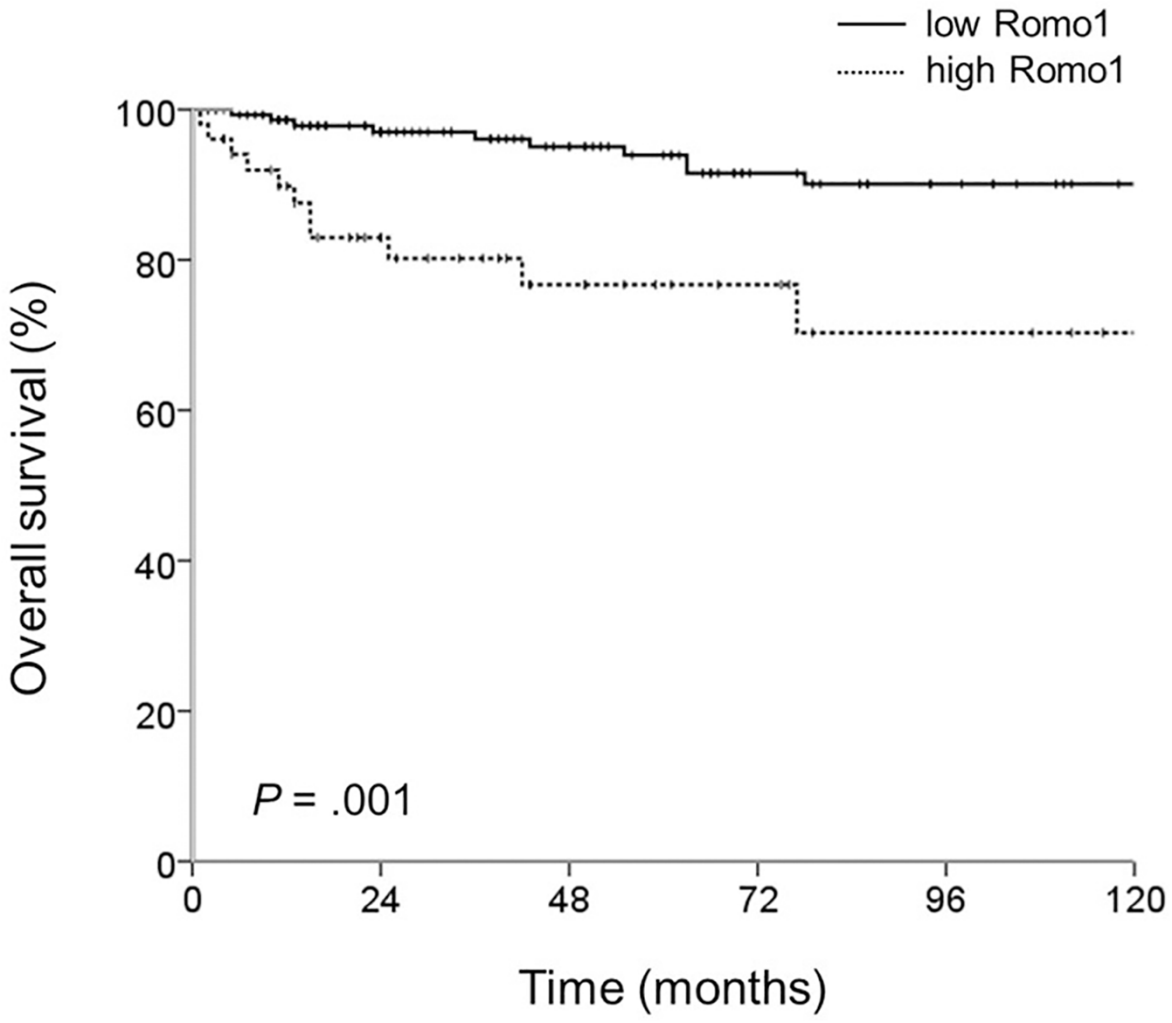
The cumulative overall survival rates of the whole cohort by Romo1 expression levels. P value was defined by the log rank test.

Survival data with respect to clinical parameters are shown in Table 4. The mean OS was 187.2 months (95% CI: 176.2–198.1). With univariate analysis, patients who had no lymph node metastasis (p = 0.002) and lower LNRs (p = 0.011) had better OS, and the high Romo1 group had poorer OS (195.8 vs 158.3 months, p = 0.001) (Fig 3). By multivariate analysis, lymph node metastasis (HR: 3.733, 95% CI: 1.520–9.169), higher LNR (HR: 2.329, 95% CI: 0.997–5.441), and higher Romo1 expression (HR: 3.198, 95% CI: 1.376–7.436) were significantly related to poorer OS.

**Table 4:** Survival analyses for the whole cohort.

	No. of patients (%)	Overall survival (OS)		
		Mean OS (months)	Univariate analysis p	Multivariate analysis adjusted HR(95% CI)
All	208(100)	187.2		
Gender			0.184	
Female	93(45)	197.6		
Male	115(55)	178.7		
Age			0.913	
<70	171(82)	187.7		
≥70	37(18)	156.2		
N stage			0.002	3.733(1.520~9.169)
N0	118(57)	200.5		
≥N1	90(43)	167.1		
LNR			0.011	2.329(0.997~5.441)
<0.1	151(73)	194.1		
≥0.1	57(27)	165.3		
T stage			0.27	
T1,T2	35(17)	195.9		
T3,T4	173(83)	185		
Tumor differentiation			0.101	
Well	85(41)	198.4		
Mod-poor	123(59)	178.4		
Romo1			0.001	3.198(1.376~7.436)
Low	154(74.03)	195.8		
High	54(25.97)	158.3		

N, lymph node; LNR, lymph node ratio; T, tumor.

### 3.6 Effects of Romo1 expression on cell proliferation

**Fig 4:**
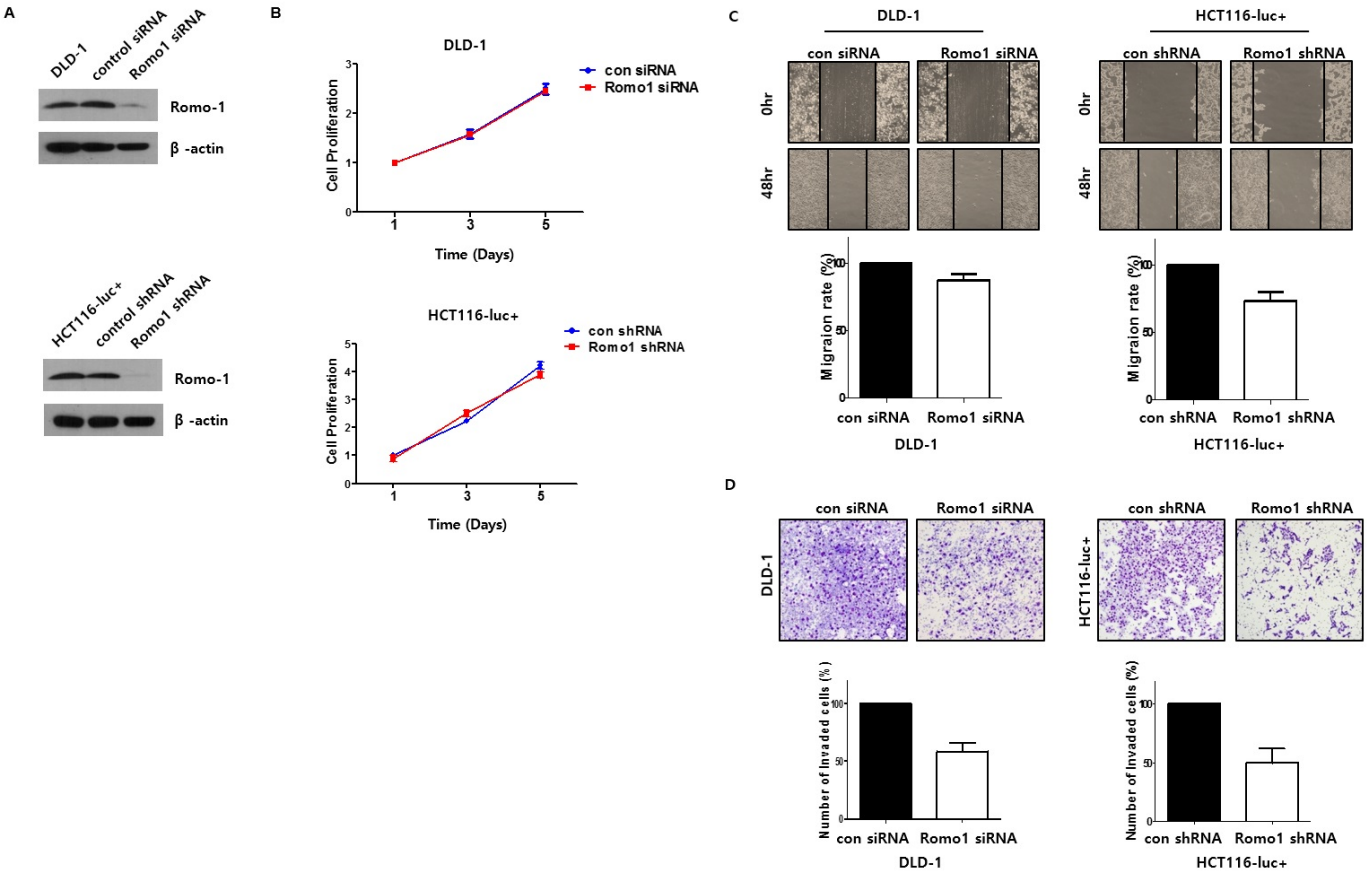
(A) Western blotting was performed to confirm knockdown of Romo1. (B) The cell viability of the colorectal cancer (CRC) cells was determined by MTT assay after transfection with Romo1 siRNA, Romo1 shRNA, control siRNA, or control shRNA. Romo1 had no effect on cell viability and proliferation in CRC cells. (C) The effects of Romo1 on cell motility in CRC cells were determined by wound healing assay. Cell monolayers were scratched with a pipette tip and incubated with 5% FBS medium. Cell migration to the wound area was then monitored for 0hr, 24hr, and 48h post-wound, and the percentage of total area covered by the cells was then assessed using the NIH Image program. (D) Matrigel invasion assay was performed to determine the effects of Romo1 on the invasive ability of CRC cells. The invaded cells on the bottom chamber were stained with crystal violet and counted. Images of invasive HCT116 and DLD-1 cells are shown.

**Fig 5:**
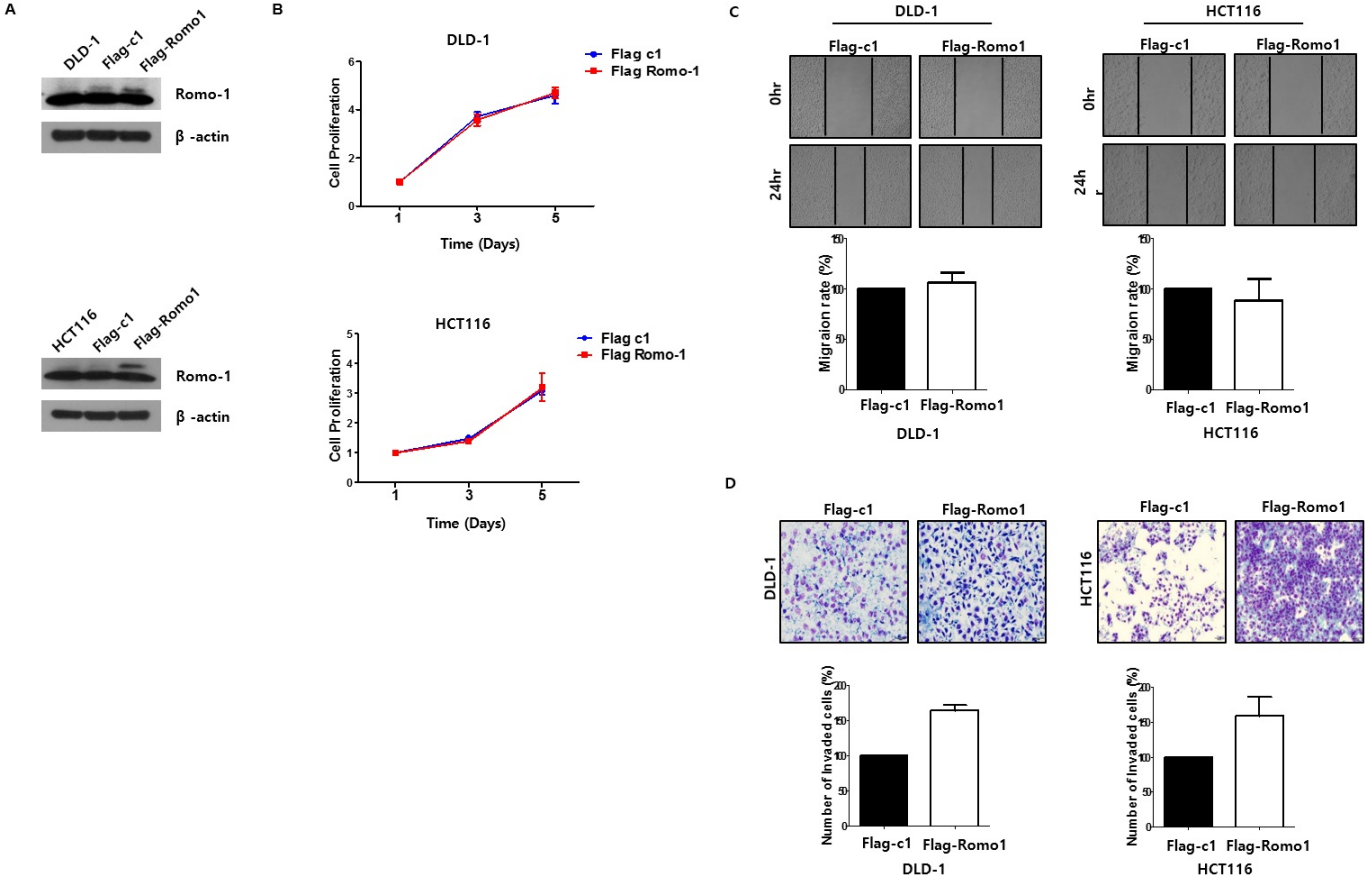
(A) Western blotting was performed to confirm upregulation of Romo1. (B) The cell viability of the colorectal cancer (CRC) cells was determined by MTT assay after transfection with pFlag-c1 or pFlag-c1 Romo1. Romo1 had no effect on cell viability and proliferation in CRC cells. (C) The effects of Romo1 on cell motility in CRC cells were determined by wound healing assay. Cell monolayers were scratched with a pipette tip and incubated with 5% FBS medium. Cell migration to the wound area was then monitored for 0hr, 24hr, and 48h post-wound, and the percentage of total area covered by the cells was then assessed using the NIH Image program. (D) Matrigel invasion assay was performed to determine the effects of Romo1 on the invasive ability of CRC cells. The invaded cells on the bottom chamber were stained with crystal violet and counted. Images of invasive HCT116 and DLD-1 cells are shown.

Even though a number of studies have reported a critical role for Romo1 in various cancers’ cell proliferation, Romo1 expression was not significantly related to tumor size or T staging in our cohort. To study the role of Romo1 in CRC cell proliferation, after we confirmed Romo1 knockdown or upregulation by western blotting (Fig 4A, Fig 5A), we performed MTT assays. There was no change in the Romo1 knockdown cells or Romo1 upregulated cells (Fig 4B, Fig 5B), which was consistent with the results of analyzing the patients’ clinical parameters.

### 3.7 Effects of Romo1 expression on invasive activity of CRC cells

In the present study, Romo1 overexpression correlated closely with higher LNR and lymphatic invasion of primary tumors, therefore, we suppose that Romo1 plays an important role in CRC metastasis, especially lymphatic metastasis. To investigate the effects of Romo1 expression on the metastatic ability of CRC cells, we performed Matrigel invasion assay. Compared with controlled CRC cells, invasive activity decreased by approximately 51% in HCT116 transfected with Romo1 shRNA and approximately 42% in DLD-1 transfected with Romo1 siRNA (Fig 4C). Compared with controlled cells, invasiveness increased by about 71% in HCT116 transfected with pFlag-c1 Romo1 and about 61% in DLD-1 transfected with pFlag-c1 Romo1 (Fig 5C). These results suggest that Romo1 acts as a key regulator of lymphatic metastasis by increasing the invasive activity of CRC cells. Next, we investigated the role of Romo1 on CRC cell motility with a wound healing assay. However, cell motility was not changed in the Romo1 knockdown cells or Romo1 upregulated cells (Fig 4D, Fig 5D).

## 4. Discussion

The current study showed that high Romo1 expression in cancer tissues is significantly related to early recurrence and poor survival in CRC patients who had curative resection (n = 190). When the patients with stage IV CRC were included, the Romo1 expression level was significantly associated with the survival of the whole cohort (n = 208). In addition, we found that Romo1 overexpression was significantly associated with higher LNR and lymphatic invasion of primary tumors as well as poorer survival in CRC patients. In order to validate that Romo1 induces high lymphatic metastatic tendency, we conducted an *in vitro* study that revealed the role of Romo1 in cancer invasiveness; specifically, knockdown of Romo1 decreased cancer cell invasion. It is thought that Romo1 induces cancer invasiveness, therefore promotes lymphatic invasion of primary tumor, which is an important process of lymphatic metastasis; Lymphatic metastasis induced by Romo1 is thought to be a key reason for the poor survival associated with Romo1 overexpression. To the best of our knowledge, this is the first study to identify Romo1 as a potential adverse prognostic marker in CRC patients and to propose increased cancer invasion as the key to the harmful effects of Romo1 in CRC.

Romo1 is reported to be a key regulator of ROS release from mitochondria[7]. Because increased ROS levels had been shown in various cancers and observed to be related with carcinogenesis and cancer progression[22–24], Romo1 had mainly been investigated in oncology fields. In a previous study, intracellular ROS levels and cancer cell growth decreased with Romo1 knockdown. This study also showed that the downregulated cell proliferation caused by Romo1 knockdown began with blocking ERK activation, which is crucial for cell cycle entry[9, 25]. It is suggested that Romo1 induced chronically increased ROS levels and that the resultant chronic oxidative stress may contribute to carcinogenesis as well as cell proliferation[9]. Another report demonstrated that Romo1 induced not only cancer cell proliferation but also cancer cell invasion via deregulated NF-kB signaling pathway by showing this pathway’s downregulation caused by Romo1 knockdown. Deregulated NF-kB is reported to contribute to cancer progression, cell proliferation, and the resistance to apoptosis of diverse tumor cells[11, 26–28]. In addition, invasive activity induced by Romo1 was shown to be blocked by inhibitor of kB kinase (IKK), demonstrating the association between Romo1 expression and the NF-kB pathway[12].

Even though a number of *in vitro* studies have been conducted in the decade since Romo1 was discovered, the clinical applications of this protein are not well-known. As previously mentioned, a few studies determined Romo1 to be a potential prognostic biomarker in patients with HCC and NSCLC[14, 16, 17], and multiple *in vitro* studies demonstrated Romo1’s role in cancer biology; therefore, we could hypothesize that Romo1 might have clinical significance in CRC. The current study shows the potential of Romo1 as a predictive biomarker in patients with CRC. Furthermore, we demonstrated an association between Romo1 overexpression and high lymphatic metastatic tendency, which has not found in previous retrospective studies. It is unclear why, despite the same results from *in vitro* studies regarding Romo1’s role in cancer invasiveness, lymphatic metastasis did not appear to relate to Romo1 overexpression in other cancer cohorts. In the current study, only lymphatic invasion of primary tumor, not vascular invasion, was shown to correlate with Romo1 expression. Both lymphatic and vascular invasion used to be discussed as comprehensive concept, lympho-vascular invasion, however, since it has been known that they have different meanings in each cancer type[29, 30], they have begun to be analyzed separately. Intravasation of primary tumor into either vasculature or lymphatics primarily occurs through the angiogenic or lymphangiogenic vessels rather than via the dormant vessels[31]. As tumor lymphangiogenesis and angiogenesis take place with different pro-angiogenic factors secreted by tumor cells[32,33], intravasation into either microvascular or lymphatic systems is thought to depend on different processes, and some studies have been demonstrated it[34]. Regarding the results from the current study that Romo1 correlates with only lymphatic invasion but not vascular invasion, we suppose that Romo1 could induce specific signals that promote lymphangiogenesis in CRC. Possible examples include VEGF-C, VEGF-D, ephrin-B2, and lymphatic vessel endothelial hyaluronan receptor 1. Because the correlations between angiogenesis and lymphangiogenesis promoted by VEGF families and ROS have been reported multiple times[35, 36] and Romo1 is an upstream ROS mediator, it would be interesting to study the role of Romo1 in lymphangiogenesis.

In this study, N stage did not seem to have a significant correlation with Romo1 expression. Considering the association of Romo1 expression with lymphatic invasion and invasive ability of CRC cells, we expected that Romo1 would be associated with the N stage, however we could not show statistically significant correlation. This finding seems to be due to the limitation of this study, which is that the number of dissected lymph nodes were less than 12 nodes in 31 of 208 patients (about 15%) resulting in underestimating N stage of patients; During the period that surgeries of this cohort were performed (1998–2000), dissecting lymph nodes more than 12 was not recommended strongly. With the exclusion of the 31 patients who had dissected lymph nodes less than 12 nodes, Romo1 scores in patients with N0 were significantly lower than those with N stage more than N1 (n = 95 and 82 respectively, p value was 0.043)(S1 Fig). Even when including the 31 patients, Romo1 was shown to be correlated with high LNR, which is reported to be superior to the previous N staging system in predicting and stratifying the prognosis of a number of cancers including CRC in recent studies[20, 21]. LNR is a parameter calculated by the number of dissected lymph nodes, and it seems the reason why Romo1 correlates only with LNR but not N stage, in this cohort. In addition although not statistically significant, the average Romo1 score of patients with N stage more than N1 was higher than that of patients with N0 (14.0 and 13.4 respectively). Since, this cohort included a small number of patients in each stage of cancer and had surgeries a long while ago (1998 to 2000), further studies on the clinical application of Romo1, including more patients and recent data, should be performed and a direct relationship between Romo1 and the N stage could be found.

In the current study, there was no significant correlation between Romo1 expression and tumor size, which was also previously found in an HCC cohort. Interestingly, the cell proliferation related to Romo1 overexpression that was demonstrated in cancers including non-small-cell lung cancer, cervical cancer, basal cell carcinoma, and HCC[9] was also not observed in the present study. Although it is unclear why Romo1 does not affect cell proliferation and tumor size in CRC unlike in other cancers, the results from both the analysis of the patients’ clinical outcomes and the *in vitro* study accorded with each other. Additional studies are needed on the different roles of Romo1 in the cell proliferation of different cancers.

Romo1 could be a target of future drug. Romo1 overexpression not only had consistently been associated with cancer invasiveness in a variety of *in vitro* studies and in a retrospective study with HCC patients[14], but also was shown to be essential in lymphatic metastasis in the current study. Since the major cause of cancer mortality is metastasis[37], the patients’ survival rate could be improved by targeting Romo1. As a matter of fact, because unwanted chronic ROS stress is thought to induce carcinogenesis and tissue invasion, various antioxidants have been used to avoid cancer invasion in a number of trials that were ultimately unsuccessful[38]. Regulating mitochondrial ROS release or production rather than eliminating ROS already produced by mitochondria could be another method of preventing cancer cell invasion, and for this reason, we suggest Romo1 as a promising target of cancer treatment. Through further study to determine the exact functions of Romo1, other favorable targets for cancer treatment might be found.

Univariate and multivariate analyses in this study showed that upregulating Romo1 was an independent risk factor regarding both OS and DFS. The function of qualified prognostic biomarkers is not only to predict recurrence or survival but also to identify patients who should be receiving specific treatments. For the last ten years, DFS in patients with stage III CRC has improved significantly with the addition of oxaliplatin to previous adjuvant chemotherapy regimens (5FU/LV)[39–41]. Because there are no definite criteria for identifying stage II CRC with a high risk of recurrence, this successful outcome was not shown in early-stage CRC[42]. Because Romo1 is found to be related to poor survival in stage I-III CRC, and interestingly, Romo1-induced ROS production was reported to be associated with acquired resistance to 5FU[43], we suppose that Romo1 could be a biomarker that can detect high-risk patients with stage II CRC who need to be treated with adjuvant chemotherapy such as oxaliplatin plus 5FU/LV. The cohort in this study had been treated before 2000, that is, before oxaliplatin had been introduced, and therefore, it was not possible to analyze Romo1’s potential as a predictive marker.

This study has several limitations. First of all, the current study was performed retrospectively at a single center. Second of all, IHC was used for quantification of Romo1 expression, which possibly has variant results. However, there is no standard quantification method established for measurement of Romo1 expression and IHC is a generally performed method for measuring protein expression. In this study, all possible cutoff levels of Romo1 were statistically analyzed, similar to previous studies on the likelihood of Romo1 as a predictive biomarker. As in previous study[17], when the cutoff was used at about the upper third of the Romo1 scores, the results were statistically significant (scores from 19 to 21). Our results should be validated by further prospective studies including many patient, using other quantification methods also.

In conclusion, Romo1 showed potential as an adverse predictive marker in CRC patients who underwent curative resection. We suppose that Romo1 induces cancer invasiveness and consequently lymphatic invasion, which is an important process of lymphatic metastasis; Lymphatic metastasis is a key reason for the poor survival associated with highly expressed Romo1. With more extensive efforts to define the exact effects and functions of Romo1, Romo1 inhibitors could be developed and used as new anti-cancer agents. Additional current studies of large populations are needed to determine other possible clinical implications of Romo1.

## Supporting information

S1 Fig With the exclusion of the patients who had dissected lymph nodes less than 12 nodes, we compared Romo1 scores in patients with N0 and those with N stage more than N1. Average Romo1 score of patients with N0 (11.91) was significantly lower than that of patients with N1 or N2 (14.21). P value was defined by Mann-Whitney test.
(TIF)

## Abbreviations

AJCC: American Joint Committee on Cancer
CRC: colorectal cancer
DFS: disease-free survival
ERK: extracellular signal-regulated kinases
EMT: epithelial-mesenchymal transition
HCC: hepatocellular carcinoma
IHC: immunohistochemical
LNR: lymph node ratio
NSCLC: non-small cell lung cancer
NF-kB: nuclear factor kappa B
OS: overall survival
Romo1: Reactive oxygen species modulator 1
ROS: reactive oxygen species
TMA: tissue microarray
VEGF: vascular endothelial growth factor
5FU/LV: 5-fluorouracil/leucovorin
