# Supplementary figures and images for: Reactive oxygen species modulator-1 (Romo1) predicts unfavorable prognosis in colorectal cancer patients
Source: PLoS One. 2017 May 4;12(5):e0176834. doi: 10.1371/journal.pone.0176834 (PMC5417558; doi:10.1371/journal.pone.0176834)

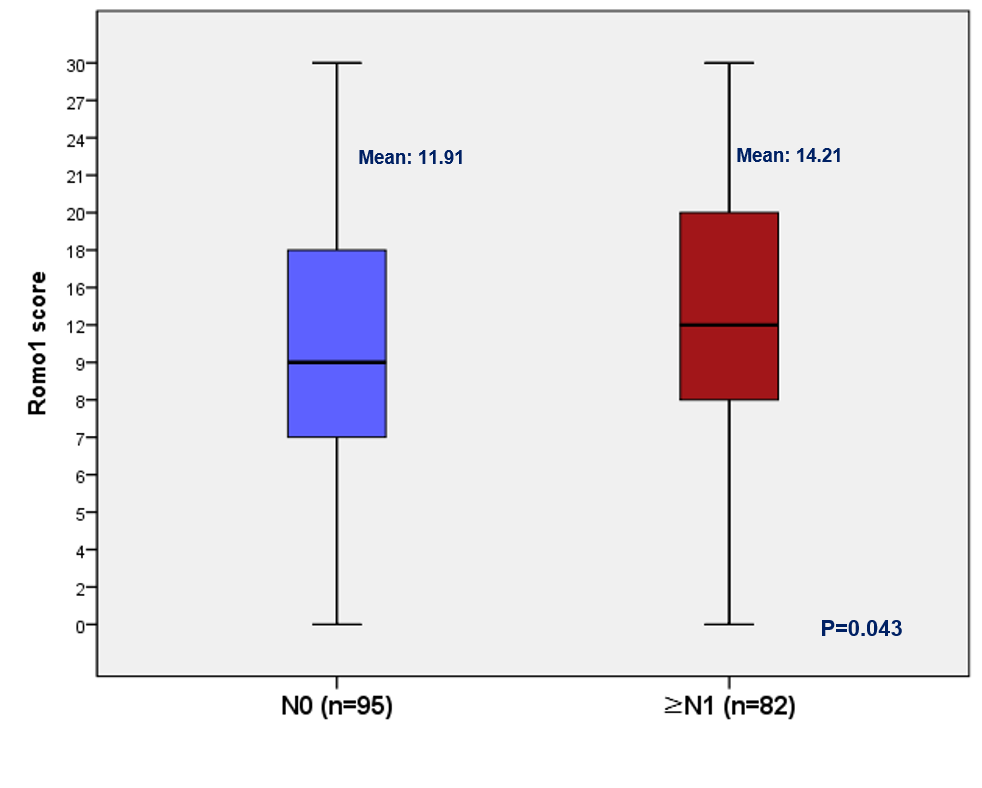

Supplement: S1 Fig — Average Romo1 score of patients with N0 (11.91) was significantly lower than that of patients with N1 or N2 (14.21). P value was defined by Mann-Whitney test. (TIF) [file pone.0176834.s001.tif]
